# Mediating the Local Oxygen-Bridge Interactions of Oxysalt/Perovskite Interface for Defect Passivation of Perovskite Photovoltaics

**DOI:** 10.1007/s40820-021-00683-7

**Published:** 2021-08-17

**Authors:** Ze Qing Lin, Hui Jun Lian, Bing Ge, Ziren Zhou, Haiyang Yuan, Yu Hou, Shuang Yang, Hua Gui Yang

**Affiliations:** Key Laboratory for Ultrafine Materials of Ministry of Education, Shanghai Engineering Research Center of Hierarchical Nanomaterials, School of Materials Science and Engineering, East China University of Science and Technology, Shanghai, 200237 People’s Republic of China

**Keywords:** Solar cell, Lead halide perovskite, Passivation, Oxysalt, Central atom

## Abstract

Passivation, as a classical surface treatment technique, has been widely accepted in start-of-the-art perovskite solar cells (PSCs) that can effectively modulate the electronic and chemical property of defective perovskite surface. The discovery of inorganic passivation compounds, such as oxysalts, has largely advanced the efficiency and lifetime of PSCs on account of its favorable electrical property and remarkable inherent stability, but a lack of deep understanding of how its local configuration affects the passivation effectiveness is a huge impediment for future interfacial molecular engineering. Here, we demonstrate the central-atom-dependent-passivation of oxysalt on perovskite surface, in which the central atoms of oxyacid anions dominate the interfacial oxygen-bridge strength. We revealed that the balance of local interactions between the central atoms of oxyacid anions (e.g., N, C, S, P, Si) and the metal cations on perovskite surface (e.g., Pb) generally determines the bond formation at oxysalt/perovskite interface, which can be understood by the bond order conservation principle. Silicate with less electronegative Si central atoms provides strong O-Pb motif and improved passivation effect, delivering a champion efficiency of 17.26% for CsPbI_2_Br solar cells. Our strategy is also universally effective in improving the device performance of several commonly used perovskite compositions.

**Electronic supplementary material:**

The online version of this article (10.1007/s40820-021-00683-7) contains supplementary material, which is available to authorized users.

## Highlights

- Oxyacid anions (NO_3_^−^, SO_4_^2−^, CO_3_^2−^, PO_4_^3−^ and SiO_3_^2−^) were investigated both theoretically and experimentally about their passivation effect on CsPbI_2_Br perovskite interface.
- Adjustment of oxysalt layer thickness can optimize the surface band position that could be beneficial for electronic band alignment at perovskite/transport layer interface.
- Using silicate as a passivator, the CsPbI_2_Br solar cells achieved a PCE of 17.26% with an open-circuit voltage of 1.36 V. This strategy is also effective for organic-inorganic perovskite solar cells.

**Electronic supplementary material:**

The online version of this article (10.1007/s40820-021-00683-7) contains supplementary material, which is available to authorized users.

## Graphical abstract

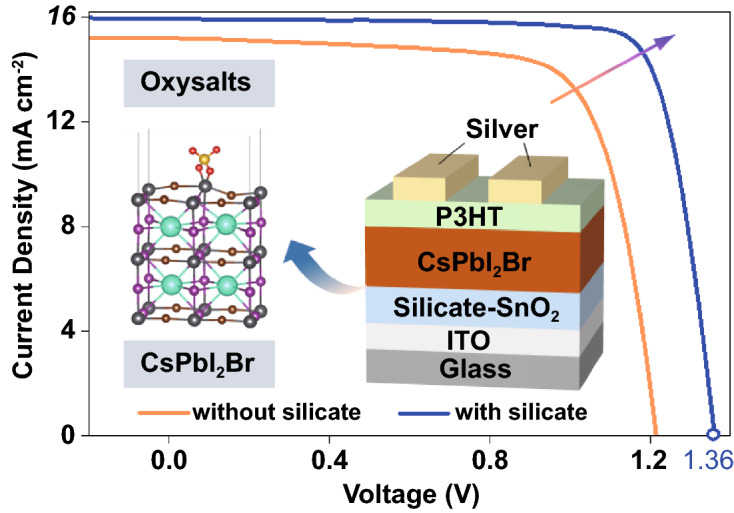

## Introduction

Lead halide perovskites have been discovered as a class of promising light-harvesting materials for photovoltaics due to their excellent optoelectronic properties including high optical absorption coefficient, adjustable bandgap and long carrier diffusion length [1–6]. During the past decade, the certificated power conversion efficiency of perovskite solar cells (PSCs) has surpassed 25%, showing a competitive performance compared to crystalline silicon solar cells [7]. Typical PSCs generally adopt the thin-film configuration comprising polycrystalline perovskite films stacked between transport layers and electrodes, whose surface or interface is crucial for device performance and longevity [8–10]. However, the surfaces or grain boundaries of perovskite films that enriched with charge trapping centers and mobile species would inevitably cause non-radiative recombination and structural decomposition, which is a major limit for the commercialization of perovskite photovoltaics [11–14].

Most often the defective perovskite surface is modified by organic functional molecules, such as ammonium halides, fullerene derivatives, zwitterions and polymers, which can passivate the charge traps by chemical interactions or charge neutralization [15–23]. Meanwhile, the perovskite community also gained inorganic passivation materials that are believed to be intrinsically more stable, which have indeed advanced the performance and stability of PSCs [24–28]. For instance, decorating the perovskite surface with a potassium halide layer can immobilize the surplus halides through complexing with potassium into benign compounds, and maximized the photoluminescence quantum yields up to 95% as well as high carrier mobility over 40 cm^2^ V^−1^ s^−1^ [29]. Most recently, Yang et al*.* reported an inorganic oxysalt interface by in situ converting perovskite surface into inorganic, strongly interacted, wide bandgap oxysalt capping layers that enabled photovoltaic devices with only 3.2% efficiency loss for 1200 h under operational condition [1]. Above-mentioned cases feature with a semiconductor-insulator interface, and particularly, the oxysalt/perovskite configuration is conceptually similar to the well-developed SiO_2_–Si passivation interface. Chemical bonding at the interface primarily determines the ultimate activity of defects and mobile sites [30, 31]. For inorganic passivation materials, their microscopic working mechanism, such as chemical bonding, electronic structures, has not yet been well resolved from both experimental and theoretical perspectives, which is urgently required for the development of this field.

In this work, through extensive modeling and experimental characterizations, we investigated the local chemical interaction of oxyacid anions (NO_3_^−^, SO_4_^2−^, CO_3_^2−^, PO_4_^3−^ and SiO_3_^2−^) on perovskite surface and revealed its central-atom-dependent-passivation phenomenon in perovskite devices. We found that the electronegativity of central atoms of oxyacid anions (X) determines the bonding strength of oxygen bridge to surface undercoordinated cations of perovskite (M) that can be interpreted by the bond order conservation principle. A less electronegative X atom, such as Si, offers a stronger local XO–M interaction and thus more reliable passivation effect. In addition, careful adjustment of oxysalt layer thickness also optimizes the surface band position that could be beneficial for electronic band alignment at perovskite/transport layer interface. These simultaneous improvements by using silicate as passivator lead to an enhanced open-circuit voltage (*V*_OC_) from 1.22 to 1.36 V and a significantly increased PCE from 13.52 to 17.26% for inorganic CsPbI_2_Br solar cells.

## Experimental Section

### Computational Details

All first-principle calculations are carried out using Vienna Ab initio Simulation Package (VASP), with the employment of the density functional theory (DFT) [32]. The exchange–correlation interactions are treated using Perdew–Burke–Ernzerhof (PBE) functional of a generalized gradient approximation (GGA) method [33]. The core–valence electron interaction was represented with the project-augmented wave (PAW) method [34]. On the plane wave basis, an energy cutoff of 450 eV is employed. The Broyden method was employed for geometric optimization until the forces on each relaxed atom were less than 0.05 eV Å^−1^. The more stable PbIBr-terminated CsPbI_2_Br (001) surface was selected in our calculation (Fig. S1), which was molded as a four layers *p*(2 × 2) with a vacuum of 20 Å in our calculation. A corresponding 1 × 1 × 1 k-point mesh was used. In the optimization, the bottom two layers were fixed, and the top two layers and the adsorbates were fully relaxed. The DFT-D3 method was used to the weak interaction [35].

### Chemicals

Cesium iodide (CsI, 99.999%), lead iodide (PbI_2_, 99.9%), lead bromide (PbBr_2_, 99.9%), dimethyl sulfoxide (DMSO, 99.8%), isopropanol (IPA,  ≥ 99.5%), bis(trifluoromethane)sulfonimide lithium salt (Li-TFSI, 99.95%), 4-tert-Butylpyridine (tBP, 96%) and toluene (TL, anhydrous, 99.8%) were purchased from Sigma-Aldrich. Tin (IV) oxide (15% in H_2_O colloidal dispersion) was purchased from Alfa-Aesar. Nickel(II) chloride hexahydrate (NiCl_2_·6H_2_O, AR, ≥ 98.0%), citric acid monohydrate (C_6_H_8_O_7_·H_2_O, AR, ≥ 99.5%), sodium sulfate (Na_2_SO_4_, AR, ≥ 99.0%), sodium carbonate (Na_2_CO_3_, AR, ≥ 99.8%), sodium nitrate (NaNO_3_, AR, ≥ 99.0%), trisodium phosphate dodecahydrate (Na_3_PO_4_·12H_2_O, AR, ≥ 98.0%), sodium metasilicate nonahydrate (Na_2_SiO_3_·9H_2_O, AR, ≥ 98.0%) were purchased from Sinopharm Chemical Reagent Co., Ltd. Formamidinium iodide (FAI) was purchased from Great Cell. Methylammonium iodide (MAI), methylammonium chloride (MACl), poly(3-hexylthiophene-2,5-diyl) (P3HT) were obtained from Xi’an Polymer Light Technology Corp. 2,2’,7,7’-tetrakis-(*N*,*N*-di-4-methoxyphenylamino)-9,9’-spirobifluorene] (spiro-OMeTAD), [6, 6]-phenyl-C_61_-butyric acid methyl ester (PC_61_BM, 99.5%) and bathocuproine (BCP, 99%) were purchased from Nichem chemicals. Fluorine-doped tin oxide (FTO) substrates (8 Ω sq^−1^) and indium-doped tin oxide (ITO) substrates (7 Ω sq^−1^) were purchased from Nippon Sheet Glass.

### Preparation of Solutions and Devices

#### Preparation of Precursor Solutions

1 mL of SnO_2_ colloidal solution (15 wt%) was firstly diluted in 5 mL of deionized water. Then, sodium salts with different anions were introduced into the diluted SnO_2_ solution with stirring at room temperature overnight. The concentration of sodium salts in SnO_2_ solution was 0.05 M. CsPbI_2_Br precursor solution was prepared by dissolving 311.77 mg CsI, 276.61 mg PbI_2_, 220.20 mg PbBr_2_ in 1 mL DMSO and stirring at 50 °C overnight. The CsPbI_2_Br precursor was filtered by a 0.2 µm polytetrafluoroethylene filter before use. P3HT solution was prepared by dissolving 15 mg P3HT in 1 mL toluene and stirring at 70 °C for 1 h. For FA-based devices, 622.35 mg PbI_2_ was dissolved in 900 μL DMF and 100 μL DMSO and stirred at 60 °C overnight before use. 94.58 mg FAI and 7.42 mg MACl were dissolved in 1 mL IPA to obtain the FAI/MACl precursor. The spiro-OMeTAD solution was prepared by mixing 80 mg spiro-OMeTAD, 54 μL Li-TFSI solution (260 mg Li-TFSI in 1 mL acetonitrile) and 11.2 μL 4-tBP in 1 mL chlorobenzene. For MA-based cells, 599.31 mg PbI_2_ was dissolved in 700 μL DMF and 300 μL DMSO and stirred at 60 °C overnight. 40 mg MAI was dissolved in 1 mL isopropanol to obtain MAI precursor.

#### Device Fabrication

We adopted a planar heterojunction structure (ITO/SnO_2_/perovskite/P3HT/Ag) in our work. Firstly, the patterned ITO substrates were washed by ultrasonication with soap, deionized water, acetone and isopropanol, respectively, for 30 min, then dried by nitrogen flow and finally treated with ultraviolet ozone cleaner for 30 min. SnO_2_ solutions were spin-coated onto the glass/ITO substrates at 3000 rpm for 30 s in ambient air, followed by annealing at 150 °C in muffle furnace with the heating rate of 2 °C min^−1^ for 30 min. After cooling to room temperature, the substrates were treated with ultraviolet ozone for 15 min and then transferred to the nitrogen-filled glovebox. Subsequently, 40 μL CsPbI_2_Br precursor was loaded onto the substrate and spin-coated via a two-step process with 1000 rpm for 10 s and 4000 rpm for 20 s. The CsPbI_2_Br layer was obtained by annealing the precursor film at 42 °C for 2 min and 160 °C for 10 min. P3HT transport layer was deposited onto the CsPbI_2_Br film by spin coating 20 μL P3HT solution at 4000 rpm for 30 s and followed by annealing at 120 °C on a hotplate. For the FA-based perovskite fabrication, two-step sequential deposition method was employed by spin coating 15 μL PbI_2_ precursor at 2000 rpm for 30 s, and then spin coating 35 μL FAI/MACl precursor on the top of the PbI_2_ film at 3500 rpm for 30 s, followed by annealing at 150 °C for 15 min. Next, 15 μL spiro-OMeTAD solution was deposited onto the FAPbI_3_ film by spin coating at 4000 rpm for 30 s. For the MA-based perovskite fabrication, 15 μL NiO_x_ precursor (50.79 mg NiCl_2_·6H_2_O and 60 mg citric acid monohydrate in 1 mL DMF) was deposited on the FTO substrates in ambient air at 4000 rpm for 60 s and then annealed at 100 °C for 10 min on a hotplate and 400 °C for 1 h in muffle furnace with the heating rate of 2 °C min^−1^ to obtain the NiO_x_ HTL. 15 μL Na_2_SiO_3_ solution (5 mg mL^−1^ in water) was spin-coated onto the NiO_x_ layer and annealed at 150 °C for 30 min in muffle furnace. Then, two-step sequential deposition method was employed to fabricate perovskite films by spin coating 15 μL PbI_2_ precursor and 35 μL MAI precursor in sequence at 3000 rpm for 30 s and 5000 rpm for 30 s, followed by annealing at 115 °C on a hotplate for 10 min. Next, 15 μL PC_61_BM (20 mg mL^−1^ in chlorobenzene) and 35 μL BCP (0.5 mg mL^−1^ in ethanol) were spin-coated on the top of the MAPbI_3_ films at 2000 rpm for 45 s, and 4000 rpm for 45 s, respectively. Ag or Au was finally thermally evaporated as a back electrode.

### Characterization

Fourier-transform infrared (FTIR) spectroscopy was measured by FTIR Nicolet 6700. Raman spectroscopy (Raman) was performed by Laser Raman InVia Reflex. The surface morphology and cross section morphology were collected by field emission scanning electron microscopy (FESEM, HITACHI S4800). X-ray diffraction (XRD) patterns were recorded with an X-ray diffractometer Bruker D8 Advance operated Cu Kα radiation. UV–vis spectra were collected using a Cary 500 UC-Vis–NIR spectrophotometer. Photoluminescence (PL) spectra were acquired at room temperature by exciting the samples deposited onto a non-conducting glass with the Fluorolog-3-p spectrophotometer under an excitation wavelength of 380 nm. Time-resolved PL experiments were performed by exciting the samples deposited onto glass substrates using the Endinburgh FLS890 spectrometer under ambient conditions. X-ray photoelectron spectroscopy (XPS, PHI5300, Mg anode, 250 W, 14 kV) was used to analyze the chemical states of the ETL layers, and the binding energy of the C 1 s peak at 284.8 eV was taken as an internal reference. Ultraviolet photoelectron spectroscopy (UPS) was recorded with He source of incident energy of 21.22 eV (He l line) in Ningbo Institute of Industrial Technology, CAS, Ningbo. The current density–voltage (J–V) curves of the photovoltaic devices were measured using a Keithley 2400 digital sourcemeter with a scan rate of 0.15 V s^−1^ under simulated AM 1.5G irradiation (Solar IV-150A, Zolix). Before each measurement, light intensity was calibrated with a standard Newport calibrated KG5-filtered Si reference cell. The external quantum efficiency (EQE) spectra were measured by a Zolix-SCS600 system, calibrated by Si reference solar cell. The electrochemical impedance spectra (EIS) were measured out using an electrochemical workstation (Parstat 2273, Princeton) in the frequency range of 1 MHz and 1 Hz under different positive bias voltages at dark conditions. The steady-state photocurrent output of the solar cell devices was measured by biasing the device at maxing power point by a Keithley 2400 digital sourcemeter. Devices were masked with a metal aperture to define the active area of 0.0625 cm^2^. To obtain the transient photovoltage (TPV) measurements, we exposed our perovskite solar cells to 0.1 sun background illumination and recorded the transit signal under attenuated laser pulse by an oscilloscope (InfiniiVision 3000 T X). The wavelength of the laser was 532 nm, and the pulse width was < 10 ns.

## Results and Discussion

### Theoretical Analysis of Interactions between Oxyacid Anions and CsPbI_2_Br

**Fig. 1:**
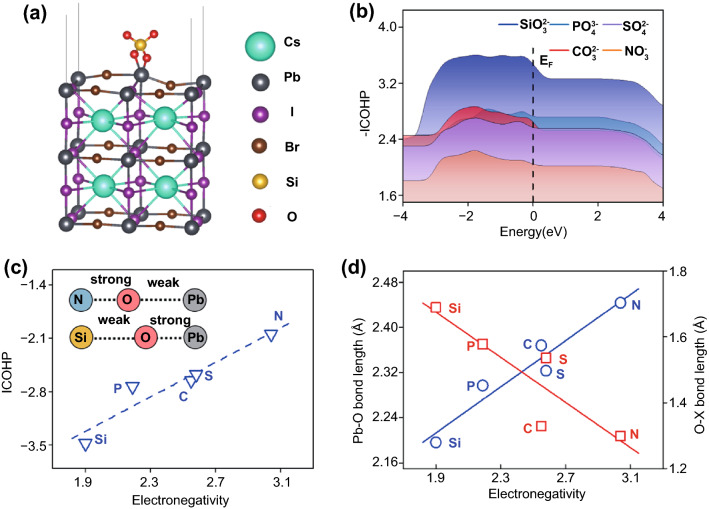
**a** Optimized structure of CsPbI_2_Br (001) surface with SiO_3_^2−^ adsorbed, where two O atoms in SiO_3_^2−^ bind with Pb site. **b** Integrated crystal orbital Hamilton population (ICOHP) between Pb site and O atom in the oxyacid anions. More negative ICOHP means the stronger bond strength of Pb–O bond. **c** Relationship for ICOHP of the Pb–O bond on CsPbI_2_Br (001) surface with different oxyacid anions adsorbed as a function of electronegativity of central element X (X = N, S, C, P, Si). **d** Relationships of the bond lengths of the X–O (X = N, S, C, P, Si) and Pb–O bonds on CsPbI_2_Br (001) surface with different oxyacid anions as function of electronegativity of central element X. There is a trade-off between the lengths of X–O and Pb–O bonds in terms of the electronegativity of central atoms

To quantitatively evaluate the interactions of different oxyacid anions (NO_3_^−^, CO_3_^2−^, SiO_3_^2−^, PO_4_^3−^, SO_4_^2−^) on perovskite, we first optimized the atomic structures of these oxyacid anions on CsPbI_2_Br perovskite (001) surface (Fig. S2), whose inorganic composition would be beneficial for experimentally isolating the surface interactions of oxysalts by spectroscopy studies. We found that the bidentate interaction structures of oxyacid anions on CsPbI_2_Br (001) are more stable, in which two bridge O atoms in an oxyacid anion bind with one Pb site (Fig. 1a). Then, we introduced the crystal orbital Hamilton population (COHP) between the Pb site and O atom on CsPbI_2_Br (001) with different oxyacid anions (Fig. S3), and their integrated COHP (ICOHP) of different Pb–O bonds were calculated by the energy integral below Femi level, which is a direct measure of the strength of the oxygen bridge to Pb sites. The more negative ICOHP means the stronger Pb–O bond, and therefore, the favored formation of XO–Pb bonds between CsPbI_2_Br and oxyacid anion. From Fig. 1b, it is clear that the interactions of these oxyacid anions with CsPbI_2_Br decrease in order of SiO_3_^2−^ > PO_4_^3−^ > CO_3_^2−^ > SO_4_^2−^ > NO_3_^−^.

We then scaled the ICOHP of different Pb–O bonds with the electronegativity of the central element X (X = C, N, Si, P, S) in different oxyacid anions and observed a linear relationship (Fig. 1c). The ICOHP of the Pb–O bond becomes more negative with the decrease in electronegativity of central atoms, meaning that the stronger interaction of oxyacid anion with CsPbI_2_Br surface. In principle, the electronegativity determines the ability of central element X to attract electrons [36]. The central element X with a smaller electronegativity would attract less electrons from the O atom in the oxyacid anion, causing a weaker X–O bond; thus, the O atom can provide more electrons to bind with cationic Pb site, giving a stronger Pb–O bond. The charge density analysis also verifies that the more electrons accumulate on the two O atoms of oxysalts as the electronegativity of the central element X is small (Fig. S4). This can also be understood by the bond order conservation principle, i.e., the less the electrons of an atom distribute over the bonds to the neighboring atom, the more each of these bonds strengthens. The bond strength changes of the X–O and Pb–O bonds on CsPbI_2_Br surface with the electronegativity are further demonstrated by the opposite change trends of the X–O and Pb–O bond lengths (Fig. 1d). Hence, the electronegativity of the central element in oxyacid anion is expected to serve as a good descriptor to assess the strength of the chemical bond between oxysalt and perovskite.

### Experimental Characterization of the Interaction between CsPbI_2_Br Perovskite and Oxysalts

**Fig. 2:**
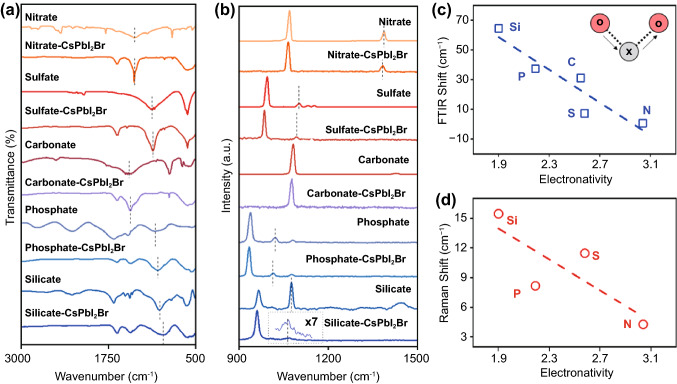
**a** FTIR spectra and **b** Raman of oxysalts powders without and with perovskite components. The asymmetric stretch (*υ*_as_) peaks of oxysalts are highlighted by dotted lines. Oxysalt and oxysalt-CsPbI_2_Br represent the pure oxysalt and the mixture of oxysalt and CsPbI_2_Br perovskite, respectively. The Raman asymmetric stretch peak of the mixture of silicate and CsPbI_2_Br is magnified 7 times. Shifts of **c** FTIR asymmetric stretch signals and **d** Raman asymmetric stretch bands of oxyacid anions by compositing with CsPbI_2_Br perovskites as a function of electronegativity. The inset image is the schematic of asymmetric stretching of O–X–O structure for oxyacid anions (X = N, S, C, P, Si)

The bonding behavior of oxysalt/perovskite interface was subsequently characterized by Fourier-transform infrared (FTIR) and Raman spectroscopy. The insensitivity of inorganic CsPbI_2_Br to vibrational spectrum made it suitable for analyzing the surface adsorption configuration of oxyacid anions. Diagnostic asymmetric stretch (*υ*_as_) of oxyacid anions generally appears at low-frequency region with the wavenumbers in the range of 900 ~ 1500 cm^−1^, while the symmetric stretch (*υ*_s_) is absent in FTIR spectra because of the unchanged dipole moment of the symmetric anions [37–46]. As shown in Fig. 2a, all the oxysalt-CsPbI_2_Br samples exhibited FTIR shift of asymmetric stretching of oxysalts compared to the pure samples without perovskite. Raman spectra of Fig. 2b also present smaller wavenumbers of *υ*_as_ for the samples with CsPbI_2_Br perovskite, in spite of the less sensitive asymmetric stretch of oxysalts. Of most significance is that the trend of *υ*_as_ shift (Δ*υ*_as_) in both FTIR and Raman spectrum exhibits a good linear relationship with respect to electronegativity values (Fig. 2c, d). The small Δ*υ*_as_ of nitrate sample spectrum indicates its unchanged configuration after contacting with perovskites, probably because of the low probability of forming O–M bond (M represents Pb or Cs). Among a serious of inorganic anions, silicate group undergoes the greatest Δ*υ*_as_ values, which again confirms the favorable chemical bonding of O atoms with Cs or Pb sites to form Si–O–M bond. As shown in Fig. S5, the *υ*_as_ band of silicate shifts from ~ 1021 cm^−1^ for pure silicate to ~ 965 cm^−1^ for silicate-PbI_2_, ~ 972 cm^−1^ for silicate-CsI and ~ 951 cm^−1^ for silicate-CsPbI_2_Br samples, respectively, which illustrate the existence of both Pb–O–Si and Cs–O–Si bonds in these samples [41]. The decrease in wavenumber in FTIR and Raman spectra implies the reduced electron density and spatial elongation of X–O bonds at the expense of bonding with Pb or Cs cations. These experimental observations are well consistent with our theoretical simulations that the weak X–O bonds can strengthen the O–Pb interaction and may subsequently lead to better surface passivation.

**Fig. 3:**
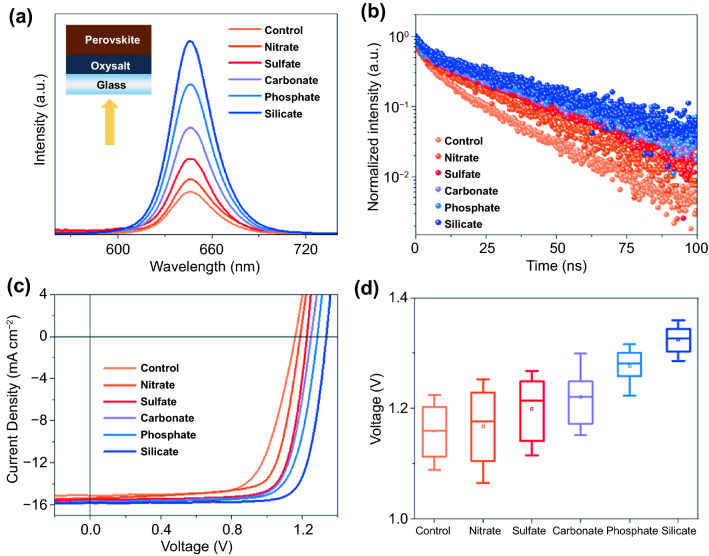
**a** Steady-state PL and **b** TRPL decay spectra of CsPbI_2_Br perovskite films without and with oxysalt passivation layer. Inset is the schematic of the PL measurement of the perovskite films. The excitation light of was irradiated from the glass side for the PL tests. **c** J–V curves and **d** *V*_OC_ distribution of CsPbI_2_Br solar cells without and with oxysalt passivation. For each kind of device, the solid transverse lines in the boxes are the average PCEs analyzed from 20 individual devices, and the error bars show the highest and lowest PCE values

To assess the electronic property of the oxysalt/perovskite interface, we measured steady-state photoluminescence (PL) and time-resolved photoluminescence (TRPL) decay of CsPbI_2_Br perovskite films deposited on oxysalt/glass substrates. A blue excitation light of 380 nm was irradiated from the glass side for all samples, which has a small penetration depth on perovskites. As shown in Fig. 3a, the PL intensity of nitrate, sulfate, carbonate, phosphate and silicate films is about 1.35, 1.78, 2.57, 3.62 and 4.71 times larger than that on bare glass, indicative of the considerably suppressed interfacial non-radiative recombination by contacting with oxysalts. TRPL curves of the samples were fitted by a biexponential equation to obtain the photocarrier lifetimes (Fig. 3b and Table S1). The fast and slow decay time constants are typically related to charge trapping process and carrier recombination process, respectively [47]. For the pristine CsPbI_2_Br film, the fast decay lifetime is 4.02 ns and the slow decay lifetime is 15.65 ns, while their fractions are 51% and 49%, respectively, highlighting the important role of charge trapping procedure of as-fabricated CsPbI_2_Br films. CsPbI_2_Br films with oxysalts passivation displayed a longer carrier lifetime compared to the pristine sample. Among all the oxysalt passivators, silicate anions improved the best and longest carrier lifetime, delivering a fast decay lifetime of 5.90 ns (38%) and a slow decay lifetime of 31.99 ns (62%). This suggests that the oxysalt passivators can effectively inhibit the presence of carrier scavengers from interfacial defects.

### Photovoltaic Performance of Oxysalt Passivated CsPbI_2_Br Devices

Solar cell devices were then fabricated with a configuration of indium tin oxide (ITO) glass /tin (IV) oxide (SnO_2_)/CsPbI_2_Br/poly(3-hexylthiophene-2,5-diyl) (P3HT)/Ag in this study (Fig. S6). To simplify the fabrication procedure of oxysalts passivated PSCs, we directly added oxysalts in the SnO_2_ colloidal aqueous solution before spin coating. The concentration of all oxysalts used in this work is 0.05 M with the molar ratio of silicate to SnO_2_ to be 1:4, if no specified. As shown in Fig. 3c and Table S2, all solar cell devices with oxysalts showed improved photovoltaic performance with the major contributor of the open-circuit voltage (*V*_OC_). In principle, *V*_OC_ is a measure of quasi-Fermi level splitting of working device that is uplifted by population of charge carriers separately in the conduction band and valence band [20]. The passivation of defective sites generally reduces the non-radiative recombination and thus produces higher density of photogenerated charges, larger quasi-Fermi level splitting as well as improved *V*_OC_ values. As expected, the average *V*_OC_ of control, nitrate, sulfate, carbonate, phosphate and silicate passivated devices is ~ 1.15, 1.18, 1.21, 1.22, 1.28 and 1.33 V, respectively (Fig. 3d). As shown in Fig. S7, the average PCE of control, nitrate, sulfate, carbonate, phosphate and silicate passivated devices is 12.66%, 13.37%, 14.04%, 14.86%, 15.60% and 16.63%, respectively, confirming the good passivation effect of oxysalts. More importantly, the PL, TRPL and J-V results again support our theoretical predications that low electronegative central atoms of oxysalts can provide better chemical bonding and defect passivation for halide perovskites. Moreover, cations may also passivate perovskite surface and deliver PCE improvement of perovskite solar cells. We then fabricated CsPbI_2_Br solar cells by using NaI, KI, Na_2_CO_3_ and K_2_CO_3_ as passivation materials. As shown in Fig. S8, we found that both Na^+^ and K^+^ improved the device performance slightly. Contrastingly, carbonate passivated CsPbI_2_Br devices showed a much more improved PCE. So, we inferred that the oxyacid anions play a critical role in improving device performance.

Opto-electrical characteristics of SnO_2_ layers and top perovskite layers should be underlying influential factors for device performance, we therefore characterized their physical and structural properties with and without silicate. Firstly, we used X-ray photoelectron spectroscopy (XPS) and X-ray diffraction (XRD) to check the existence of sodium silicate in SnO_2_ films. Obviously, Na and Si signals were observed from the composite film, as shown in Fig. S9. In contrast, the pristine SnO_2_ film showed no Na and Si signals. As shown in Fig. S10, diffraction peaks of Na_2_SiO_3_ were observed for the silicate-SnO_2_ sample, verifying the crystalline phase of solid-state Na_2_SiO_3_ (PDF# 16–0818). As expected, both SnO_2_ layers have uniform morphology and high transmittance in the visible region on ITO substrates (Figs. S11 and S12). In an effort to assess the morphology of as-prepared SnO_2_ films, atomic force microscope (AFM) imaging was performed. As shown in Fig. S13, both films exhibited a smooth surface but the roughness of the silicate-SnO_2_ film (5.95 nm) is larger than that (1.72 nm) of the SnO_2_ film. To evaluate the electrical conductivity of these films, we fabricated devices structured as ITO/SnO_2_/Ag and ITO/Silicate-SnO_2_/Ag and measured the dark J-V curves. In order to avoid the probably direct contact between Ag and ITO, we improved the films thickness of SnO_2_ layers by spin coating the precursor solution for six cycles. As illustrated in Fig. S14, rectifying behaviors were observed for both ITO/SnO_2_/Ag and ITO/Silicate-SnO_2_/Ag devices. The conductivity of silicate-SnO_2_ film was observed to be closed to that of pristine SnO_2_ film, confirming that the introduction of silicate did not affect the electrical conductivity of SnO_2_ films. After deposition of CsPbI_2_Br films, strong (100) and (200) diffraction peaks appear for both samples as shown in the XRD patterns of Fig. S15 [28]. In addition, scanning electronic microscopy (SEM) images in Fig. S16 show the similar compact and uniform morphology of CsPbI_2_Br films on both substrates. Therefore, the photovoltaic performance enhancement should be attributed to the interfacial passivation rather than the structural change of SnO_2_ and perovskite layer.

**Fig. 4:**
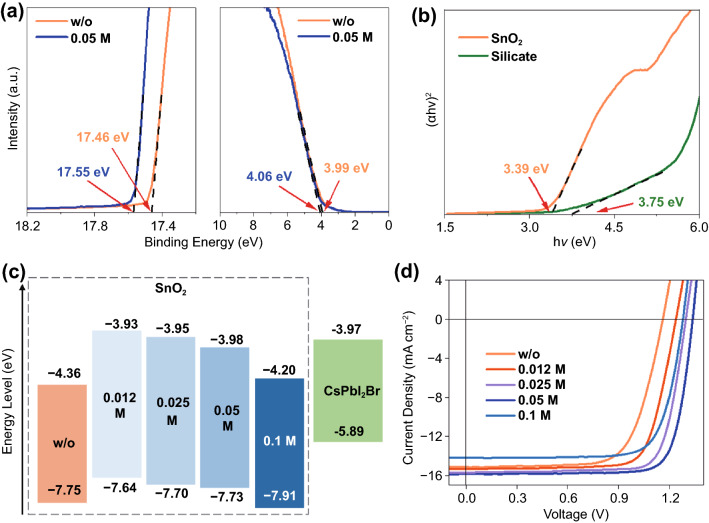
**a** Photoemission cutoff spectra (left panel) and valence band (VB) structure (right panel) of SnO_2_ films without and with silicate. The dotted lines indicate the secondary electron cutoff position and the valance band onset of the films obtained by linear extrapolating the binding energy edge to the baseline. **b** Tauc plots of the pure SnO_2_ and silicate powders. **c** Energy level scheme of the charge transport and perovskite layers in this work. **d** J–V curves of typical CsPbI_2_Br solar cells with different concentrations of silicate anions in SnO_2_ layers

The electronic band structure of SnO_2_ films with silicate was investigated by ultraviolet photoelectron spectroscopy (UPS). Figures 4a and S17 depict the secondary electron cutoff and the valence band region of UPS spectra for SnO_2_ layers without and with silicate. The concentration of silicate anions in SnO_2_ solution was varied from 0.012 to 0.1 M. By increasing the content of silicate, the valance band maximum (VBM) of SnO_2_ layers initially upshifts from − 7.75 to − 7.64 eV for 0.012 M sample and then gradually downshifts to − 7.91 eV at 0.1 M of silicate. The optical bandgap (*E*_g_) of SnO_2_ and sodium silicate was determined to be 3.39 and 3.75 eV by UV–vis spectra, respectively (Fig. 4b). For the 0.05 M sample, the molar ratio of Si to Sn is ~ 1:4, suggesting that a silicate shell should be favored on tin oxide particles. The thickening of silicate layers is likely to uplift the conduction band minimum (CBM) gradually to the value of bulk sodium silicate itself and better align the band structure of SnO_2_/perovskite interface at certain oxysalt contents (Fig. 4c) [48, 49]. PSCs with varied concentration of silicate were also fabricated, and the J–V results are shown in Fig. 4d and Table S3. The addition of silicate persistently improves the *V*_OC_ of PSCs with the silicate concentration up to 0.1 M, whereas excess oxysalts would give rise to very thick silicate shell, impede charge collection and result in low short-circuit current density (*J*_SC_) values.

**Fig. 5:**
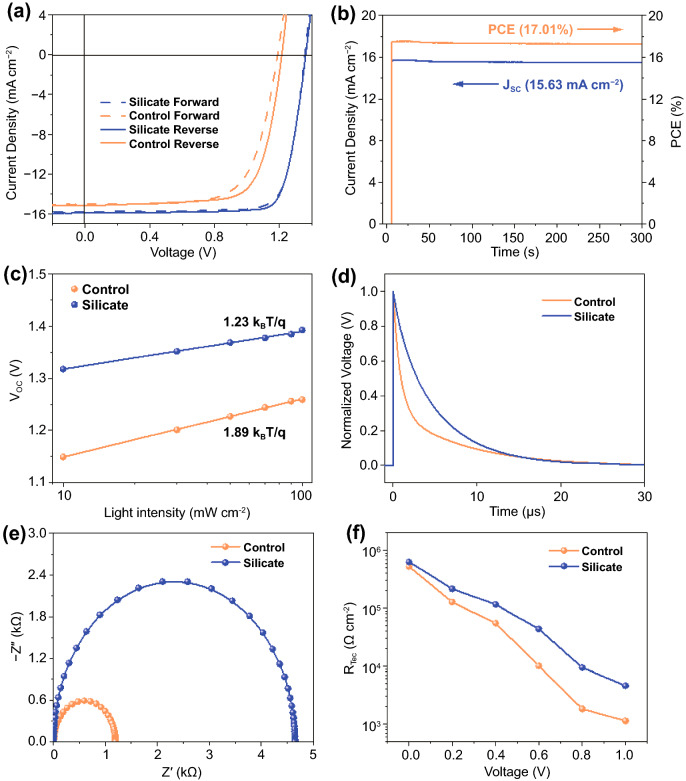
**a** J–V curves of champion PSC devices measured under simulated AM 1.5G irradiation at different scan directions. **b** Steady-state power output (blue) and current density (red) of the champion cell measured at maximum power point (MPP) voltage as a function of time. **c** *V*_OC_ values of the control and silicate passivated CsPbI_2_Br device as a function of light intensity. **d** Transient photovoltage measurements for control and silicate passivated PSCs. **e** Nyquist plots of control and silicate passivated CsPbI_2_Br devices under dark with a bias voltage of 1.0 V. **f** Dependence of *R*_rec_ on applied bias voltage for CsPbI_2_Br device with and without silicate passivation. *R*_rec_ was obtained by fitting the EIS spectra at different voltages

After optimization of experimental parameters, the champion silicate devices based on CsPbI_2_Br perovskite yielded a high PCE of 17.26% with a *J*_SC_ of 15.86 mA cm^−2^, an open-circuit voltage (*V*_OC_) of 1.36 V and a fill factor (FF) of 0.80 (Fig. 5a). In contrast, the control device without silicate delivered a PCE of 13.52% with a J_SC_ of 15.20 mA cm^−2^, an open-circuit voltage (*V*_OC_) of 1.22 V and a fill factor (FF) of 0.73. The J_SC_ values from J-V tests match well with the integrated *J*_SC_ from the external quantum efficiency spectra (EQE, Fig. S18). To the best of our knowledge, our device performance is among the highest PCEs for CsPbI_2_Br solar cells reported to date (Table S4). Noteworthy, silicate passivated device undergoes negligible hysteresis under different scan directions, suggesting reduced electronic trap states or ion migration at the interface. In addition, we also monitored the stabilized power output of our champion device under maximum power point (MPP) condition. As shown in Fig. 5b, a stabilized PCE of 17.01% was obtained together with a J_SC_ of 15.63 mA cm^−2^ for nearly 300 s under a bias of 1.09 V. The reproducibility of PSC devices was evaluated by 20 individual devices for each sample that the average PCE boosted from 12.66 ± 0.49% for the control device to 16.63 ± 0.42% (Fig. S19).

Charge recombination behavior of the PSC devices was then evaluated by light intensity-dependent *V*_OC_ measurement. As shown in Fig. 5c, the slope (kT/q) of *V*_OC_ versus the natural logarithm of light intensity for the control and silicate devices was estimated to be 1.89 and 1.23 kT/q, respectively. This discrepancy indicates that the trap-induced charge recombination was effectively suppressed in the silicate device [50]. Transient photovoltage (TPV) curves of CsPbI_2_Br solar cells were recorded by oscilloscope under the excitation of an attenuated laser pulses. The carrier recombination lifetimes of control and silicate devices are 1.7 and 4.4 μs, respectively, again confirming the suppressed charge recombination rate in devices. To further evaluate the interfacial carrier dynamic properties in CsPbI_2_Br devices, electrochemical impedance spectroscopy (EIS) was carried under different bias voltages in dark condition [51]. Figure 5e presented the Nyquist plots of control and silicate CsPbI_2_Br solar cells. The recombination resistance (*R*_rec_) of the silicate passivated CsPbI_2_Br solar cell is about 4 times larger than that of the SnO_2_-based CsPbI_2_Br device at the bias voltage of 1.0 V. After fitting the measured data under different bias voltages, it is clear that the silicate passivated devices exhibited higher *R*_rec_ value than that of the control one, indicating its slower recombination rate (Fig. 5f). We further estimated the trap density of electron-only device structured as ITO/SnO_2_/CsPbI_2_Br/PCBM/Ag by space-charge-limited-current (SCLC) measurements (Fig. S20). The trap density (*N*_defects_) is calculated by Eq. 1:

1 $$ N_{{{\text{defects}}}} = \frac{{2\varepsilon \varepsilon_{0} V_{{{\text{TFL}}}} }}{{eL^{2} }} $$

(where e is the electron charge, *L* is the thickness of the perovskite layer, *ε*_0_ and *ε* are the vacuum permittivity and the relative dielectric constant, and *V*_TFL_ is the onset voltage of the trap-filled limited region [52]). The defect density of the control and silicate passivated perovskite films was calculated to be 3.03 × 10^16^ and 2.09 × 10^16^ cm^−3^ s, respectively, which should be derived from the defect passivation of perovskite/ETL interface.

### Device Stability Study

Finally, we evaluated the long-term device stability with a device structure of ITO/SnO_2_/CsPbI_2_Br/P3HT/Au against light illumination, heat and humidity. We monitored the operational stability of the CsPbI_2_Br solar cells under continuous AM 1.5G illumination in a nitrogen-filled glovebox (Fig. S21). The efficiency of control device dropped rapidly to ~ 60% of its initial value after 500 h, whereas the silicate passivated device still retained ~ 92% of its initial value. The thermal stability of the CsPbI_2_Br solar cells was recorded by heating the devices at 85 °C in a N_2_-filled glovebox (Fig. S22). Compared to the nearly 30% loss of PCE for the control device, the silicate passivated CsPbI_2_Br solar cells maintained ~ 90% of its initial efficiency after 240 h. As illustrated in Fig. S23, the moisture stability of the silicate passivated CsPbI_2_Br solar cells was also improved that the retained PCEs of control and silicate device are ~ 50% and ~ 87%, respectively, after stored in ambient air with 15 ± 3% humidity for 1440 h. The passivation of defective surface may suppress the mass transport of perovskites and impose instructive effect on device durability.

## Conclusions

In summary, we have systematically investigated the central atom effect of inorganic oxysalts on the passivation of lead halide perovskite and elucidated the central-atom-dependent-passivation mechanism that local the interactions between O from oxysalt and metal cations from perovskite are negatively correlated with the electronegativity of central atoms. Such phenomenon can be unambiguously described by the bond order conservation principle. By using silicate passivation, we achieved a high PCE of 17.26% for CsPbI_2_Br solar cell devices, which is among the best of this class of devices. We also applied such silicate passivation strategy into FAPbI_3_ and MAPbI_3_ devices (Figs. S24 and S25). Obviously, the FAPbI_3_-based devices exhibited an improved PCE from 18.20 to 21.62%, and the MAPbI_3_-based devices showed an improved PCE from 17.50 to 20.39%, corroborating the versatility of our strategy in organic–inorganic perovskite systems (Table 1). Our findings shed light on the basic understanding about the chemical adsorption and bond formation of perovskite surface and also provide the guidelines for designing functional interface materials toward the prosperous optoelectronic application of perovskite devices.

**Table 1:** Photovoltaic parameters of the champion PSCs based on different perovskite compositions

Composition	Passivation	*J*_SC_ (mA cm^−2^)	*V*_OC_ (V)	FF	PCE (%)
CsPbI_2_Br	w/o	15.20	1.22	0.73	13.52
	Silicate	15.86	1.36	0.80	17.26
FAPbI_3_	w/o	23.71	1.07	0.71	18.20
	Silicate	24.14	1.13	0.79	21.62
MAPbI_3_	w/o	21.99	1.09	0.73	17.50
	Silicate	22.36	1.14	0.80	20.39

Devices were measured under simulated AM 1.5G irradiation at the reverse scan

## Electronic supplementary material

Below is the link to the electronic supplementary material.

Supplementary file1 (PDF 1050 kb)
