# Supplementary material for: Mediating the Local Oxygen-Bridge Interactions of Oxysalt/Perovskite Interface for Defect Passivation of Perovskite Photovoltaics
Source: Nanomicro Lett. 2021 Aug 17;13:177. doi: 10.1007/s40820-021-00683-7 (PMC8371073; doi:10.1007/s40820-021-00683-7)
Supplement: Supplementary file 1 — Supplementary file1 (PDF 1050 kb) [file 40820_2021_683_MOESM1_ESM.pdf]

Supporting Information for

Mediating the Local Oxygen-Bridge Interactions of Oxysalt/Perovskite Interface for Defect Passivation of Perovskite Photovoltaics

Ze Qing Lin[1, #], Hui Jun Lian[1, #], Bing Ge[1], Ziren Zhou[1], Haiyang Yuan[1, *], Yu Hou, Shuang Yang[1, *], Hua Gui Yang[1]

[1]Key Laboratory for Ultrafine Materials of Ministry of Education, Shanghai Engineering Research Center of Hierarchical Nanomaterials, School of Materials Science and Engineering, East China University of Science and Technology, Shanghai 200237, P. R. China

#Ze Qing Lin and Hui Jun Lian contributed equally to this work.

*Corresponding authors. E-mail: hyyuan@ecust.edu.cn (Haiyang Yuan), syang@ecust.edu.cn (Shuang Yang)

Supplementary Tables and Figures

The (001) surface of $CsPbI_2Br$ is one of the thermodynamically most stable surfaces [S1], thus which is selected in our calculations. Considering the complexity and different terminal properties of $CsPbI_2Br$ surface, we investigated the possibilities of different terminated $CsPbI_2Br$ (001), i.e., PbIBr termination and CsI termination, by building the seven layers of $CsPbI_2Br$ (001) supercell with 20 Å vacuum region (Fig. S1). The surface energies (γ) of all slab were calculated as the formula [S2]:

$$\gamma = (E\text{surf} - N{*}E\text{bulk} + \sum ni{*}\mu i)/2A$$

where E_{surf}, E_{bulk} are the energies of PbIBr or CsI-terminated $CsPbI_2Br$ (001) surface, bulk $CsPbI_2Br$, respectively. N is the number of sufficient bulk $CsPbI_2Br$ units, μi is the chemical potential, and A is the area of the $CsPbI_2Br$ (001). The more negative γ means the better stability of the surface. The calculated results show that γ of the PbIBr-terminated $CsPbI_2Br$ (001) is more negative than that of CsI-terminated one (-0.43 eV *versus* -0.34 eV). This means that PbIBr-terminal $CsPbI_2Br$ (001) is more stable and easier to be exposed during preparation, which could be the main exposed termination of $CsPbI_2Br$ (001). Hence, in our calculations, the PbIBr-terminal $CsPbI_2Br$ (001) was selected as our model.

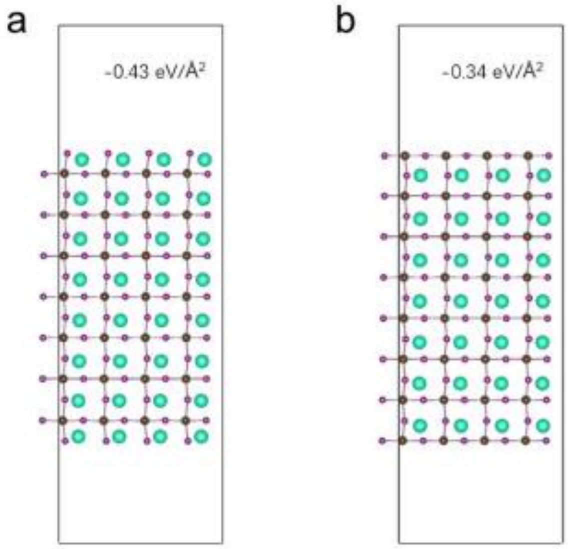

Fig. S1 Optimized $CsPbI_2Br$ (001) supercell models with (**a**) CsI-terminated and (**b**) PbIBr-terminated

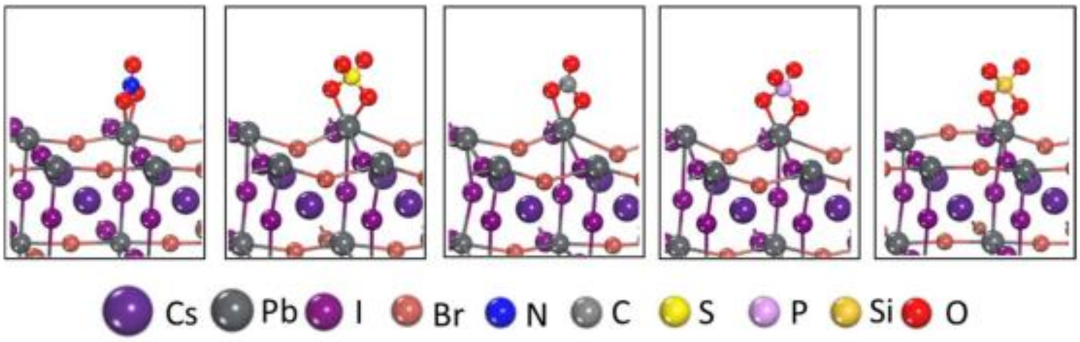

Fig. S2 Atomic structure of optimized CsPbI2Br (001) surface with oxyacid anions with different central atoms

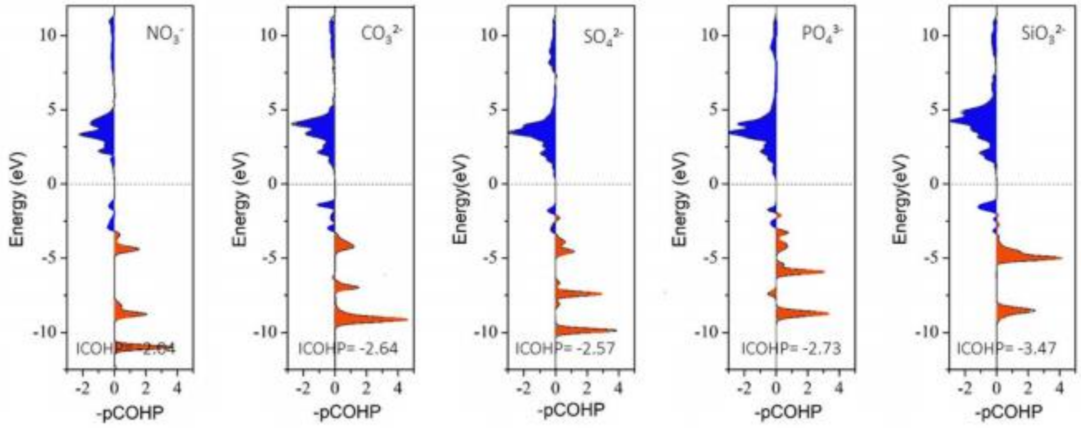

Fig. S3 Projected crystal orbital Hamilton population (pCOHP) between Pb and O atom in different oxyacid anions. The values of ICOHP calculated by the energy integral below Femi level indicated the interaction between silicate and Pb site is the strongest

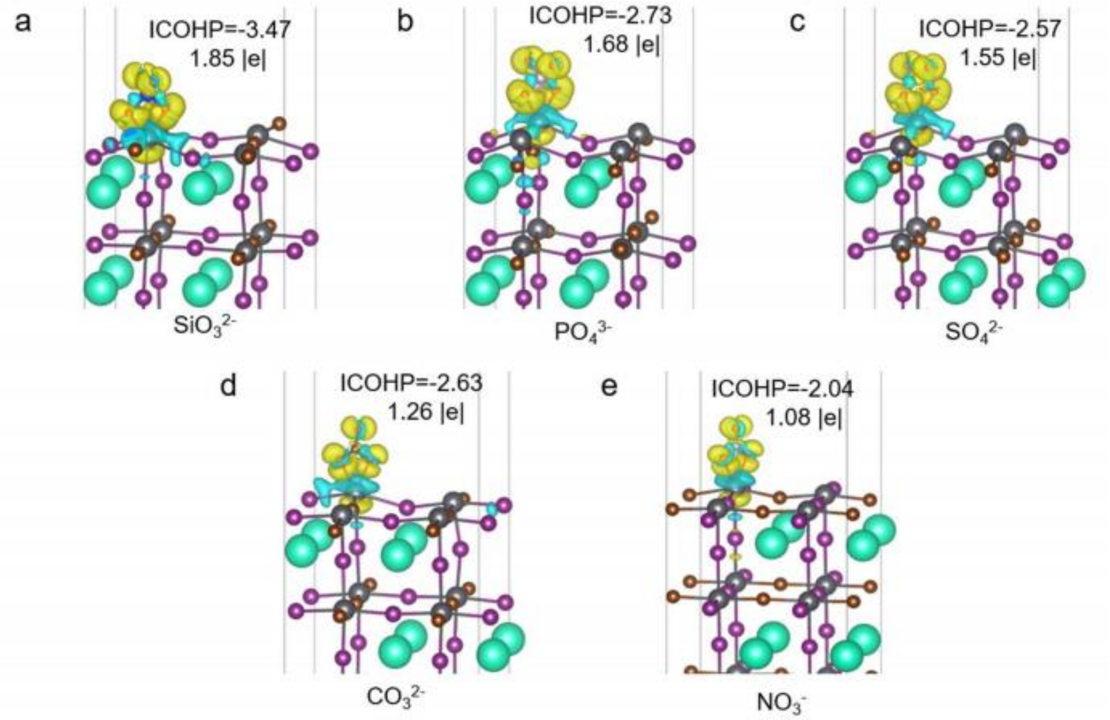

Fig. S4 Charge density difference of oxysalt/$CsPbI_2Br$ interface: (**a**) SiO_3^{2-}, (**b**) PO_4^{3-} , (**c**) SO_4^{2-}, (**d**) CO_3^{2-} and (**e**) NO^{3-}

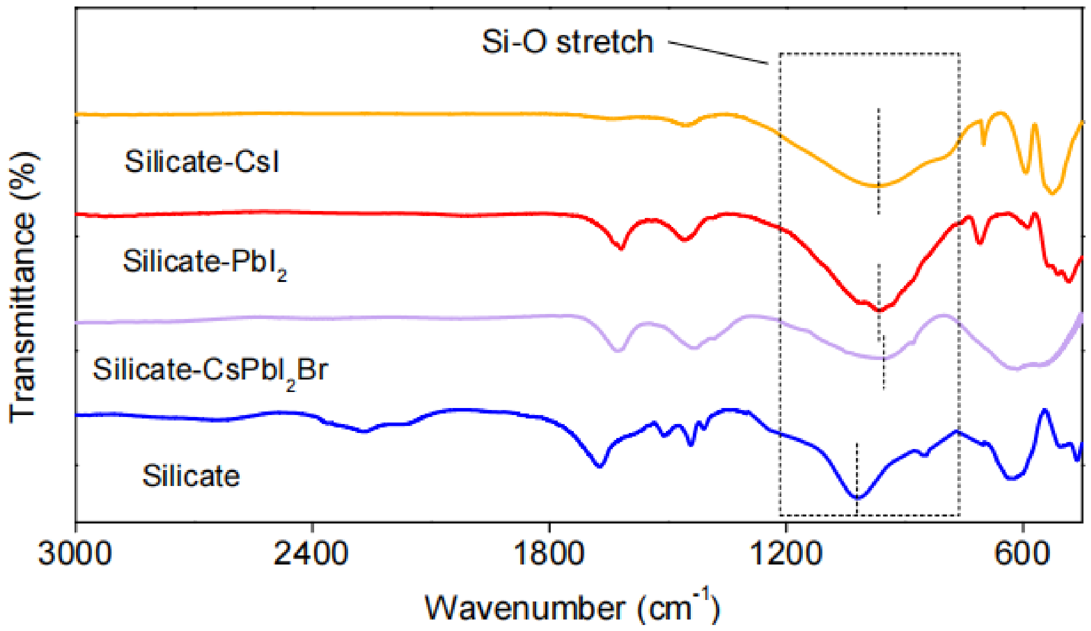

Fig. S5 FTIR spectra of sodium silicate powders without and with perovskite components. The asymmetric stretch (υ_{as}) peaks of silicate are highlighted by dotted lines

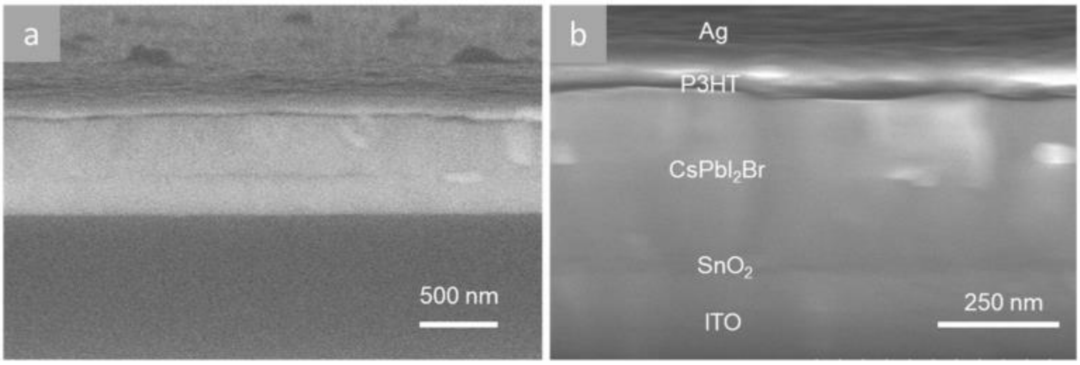

Fig. S6 Cross-sectional SEM of a typical $CsPbI_2Br$ perovskite solar cell. The device structure adopted in this work is indium tin oxide (ITO) glass/tin (IV) oxide (SnO_2)/$CsPbI_2Br$/poly(3-hexylthiophene-2,5-diyl) (P3HT)/Ag

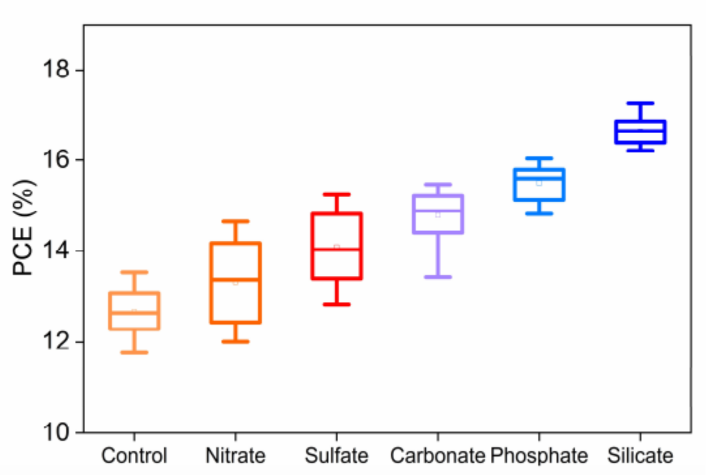

Fig. S7 PCE distributions of CsPbI2Br solar cells without and with oxysalt passivation

For each kind of device, the solid transverse lines in the boxes are the average PCEs, analysed from 20 devices, and the error bars show the highest and lowest PCE values.

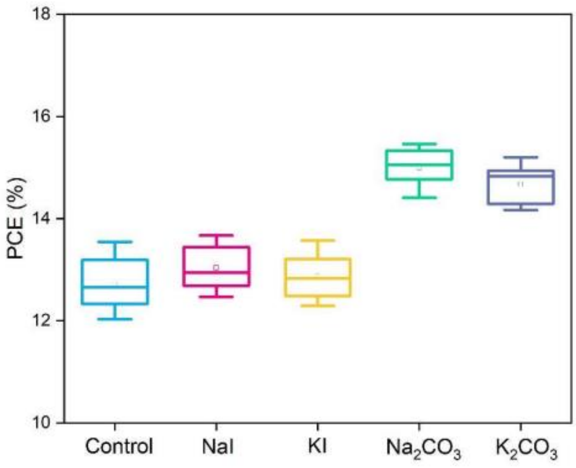

Fig. S8 PCE distributions of $CsPbI_2Br$ solar cells without and with different passivation materials

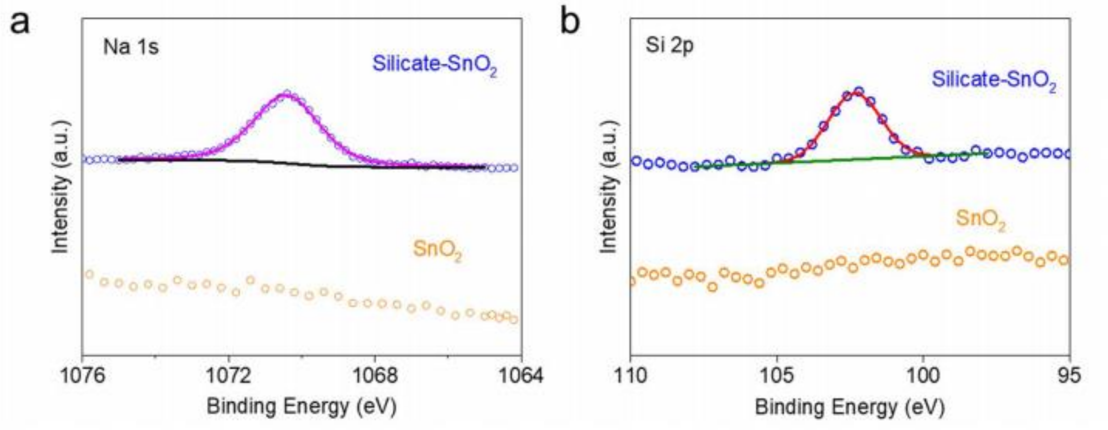

Fig. S9 (**a**) Na 1s and (**b**) Si 2p XPS spectra of SnO_2 and silicate-SnO_2 films

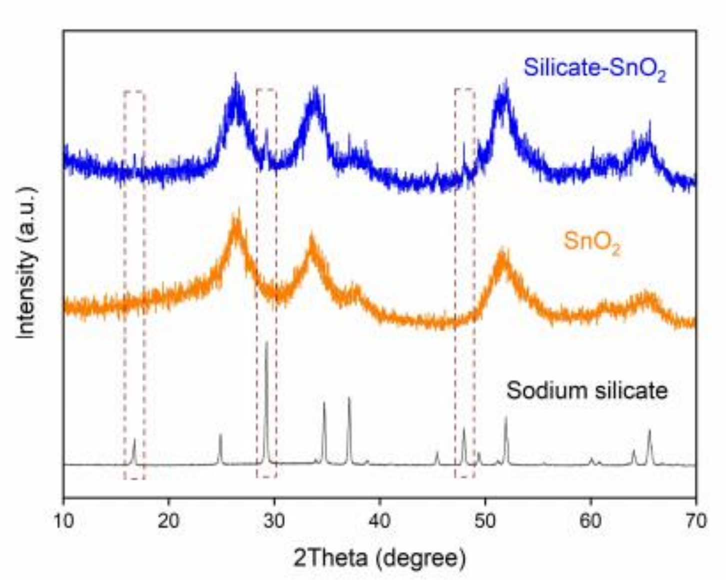

Fig. S10 XRD patterns of sodium silicate, SnO_2 and silicate-SnO_2 powders. SnO_2 and silicate-SnO_2 powders were scratched from the as-casted films. The concentration of sodium silicate in precursor solution was 0.1 M

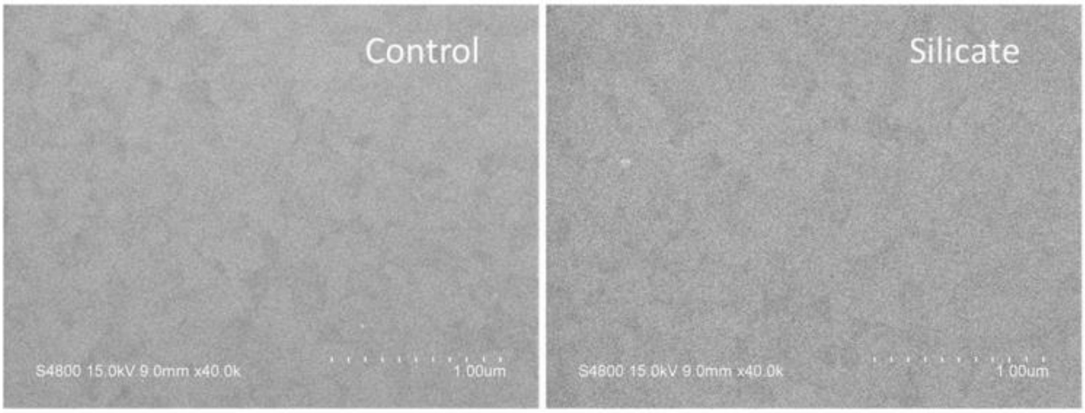

Fig. S11 SEM images of SnO_2 films with and without silicate. Both films exhibit uniform morphology with full coverage of the ITO glass substrate

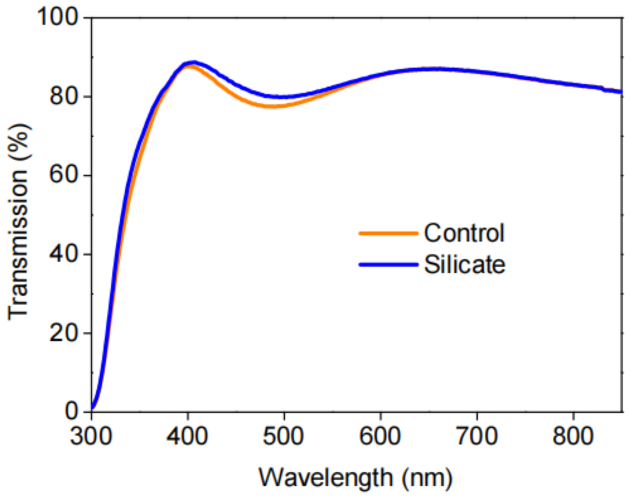

Fig. S12 Transmission spectra of SnO_2 films without and with silicate. The optical transmittance of both samples is similar in the UV and visible region.

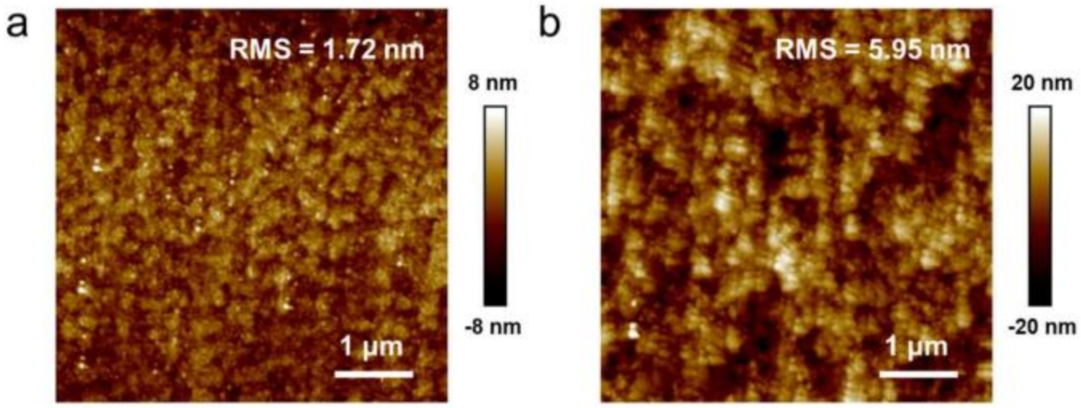

Fig. S13 AFM images of (**a**) SnO_2 and (**b**) silicate-SnO_2 films with an area of 5×5 μm^2. RMS represents root-mean-square roughness of the film

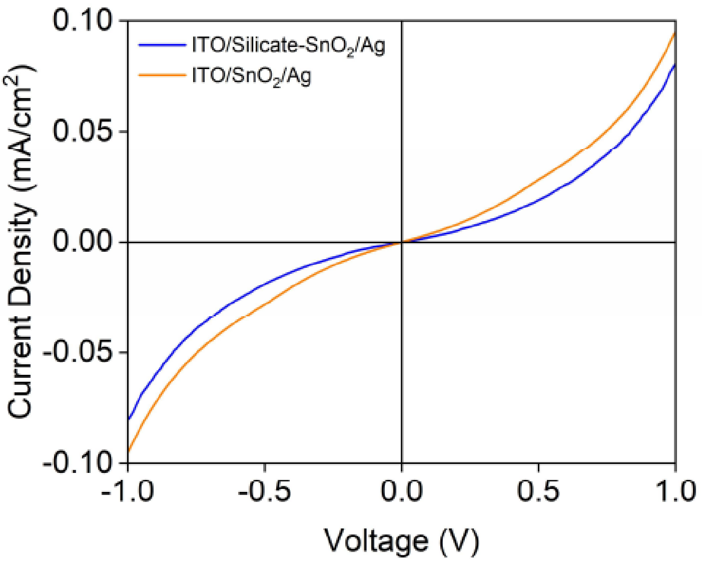

Fig. S14 Dark *J-V* characteristics of ITO/SnO2/Ag and ITO/silicate-SnO_2/Ag. Both films were spin-coated for 6 cycles to increase the film thickness.

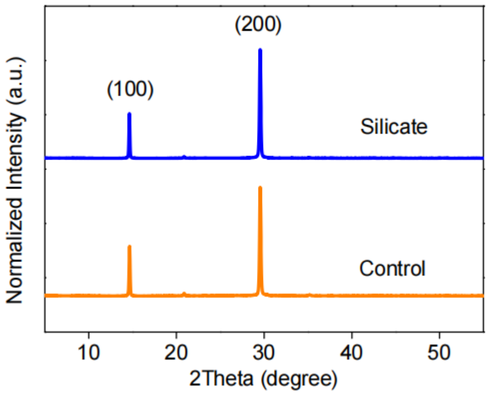

Fig. S15 XRD patterns of $CsPbI_2Br$ films deposited on SnO_2 films without and with silicate. The structure of $CsPbI_2Br$ films deposited on both SnO_2 films can be indexed as cubic perovskite structure with preferred [100] orientation.

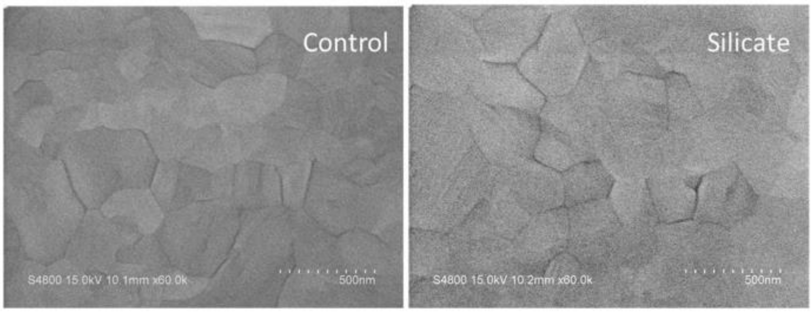

Fig. S16 SEM images of perovskites films deposited on SnO_2 films without and with silicate. Both $CsPbI_2Br$ films have compact morphology with similar grain size

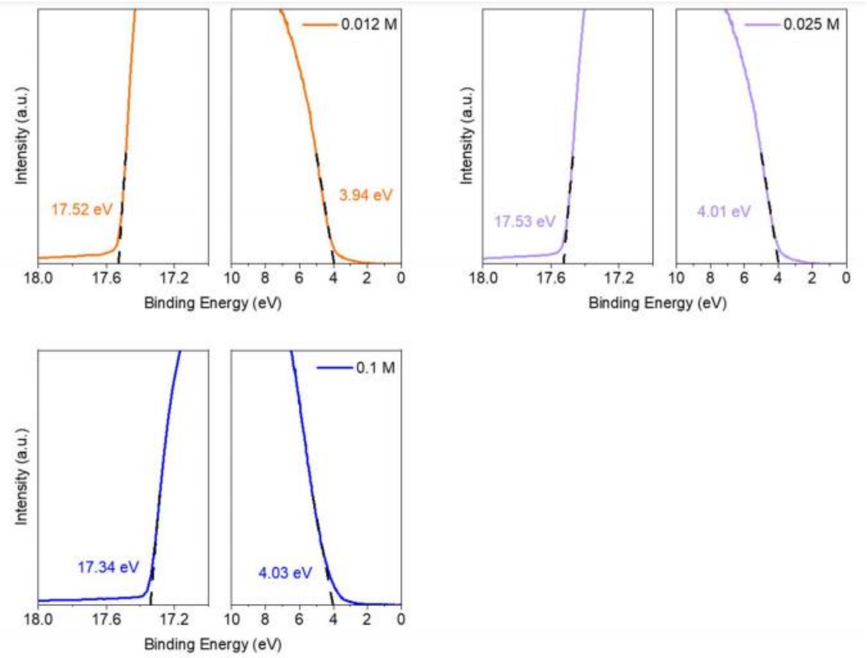

Fig. S17 UPS spectra of the SnO_2 film with a varied concentration of silicate salts. The dotted lines indicate the secondary electron cutoff position and the valance band onset of the films obtained by linear extrapolating the binding energy edge to the baseline.

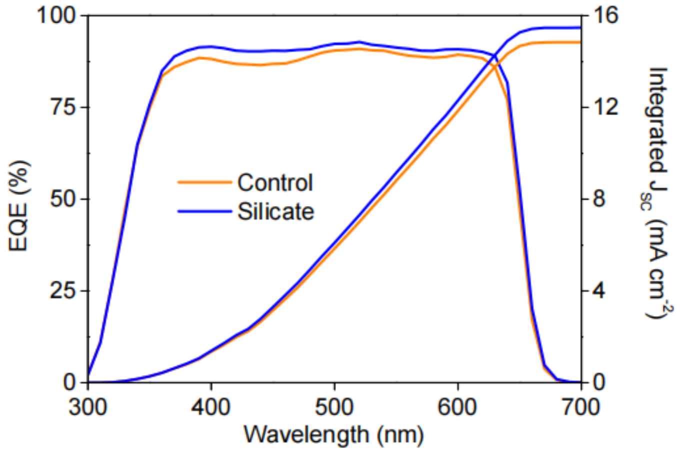

Fig. S18 EQE spectra and integrated short-circuit current density of the control and silicate passivated devices

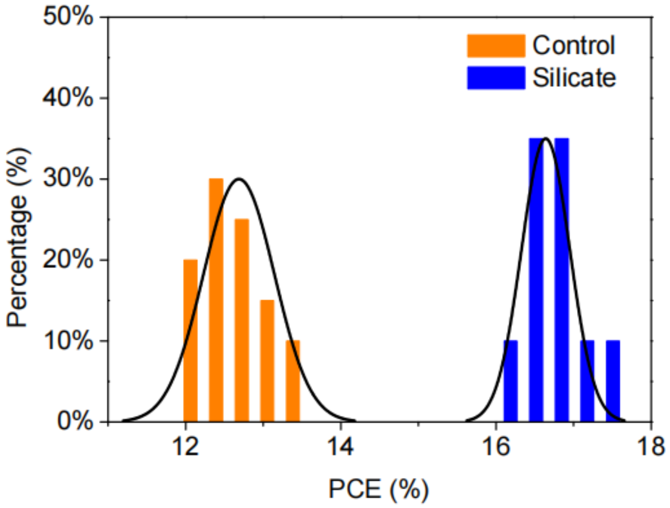

Fig. S19 Statistics of PCE distribution for control and silicate passivated PSC devices. For each type of device, the average PCE values are obtained from 20 individual cells.

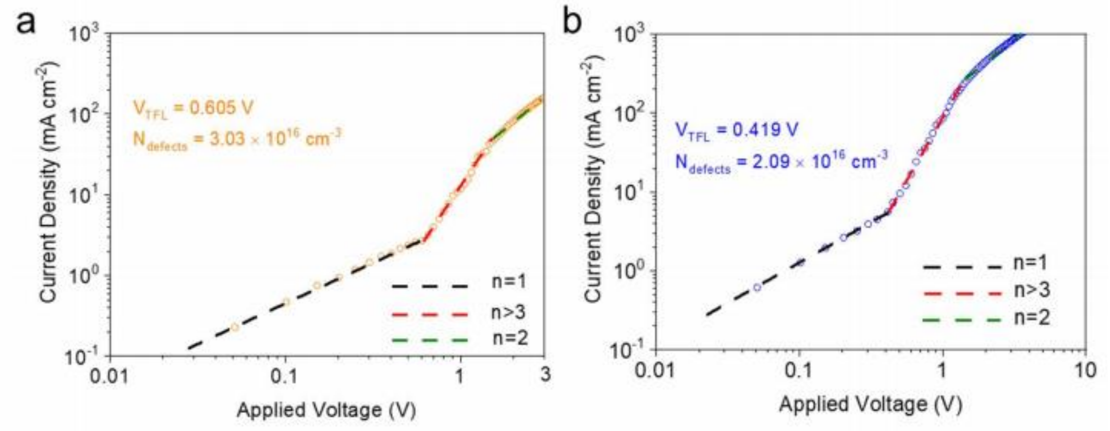

Fig. S20 SCLC measurements of electron-only perovskite devices **a** without and **b** with silicate passivation. The device structure is ITO/SnO_2/$CsPbI_2Br$/PCBM/Ag. The measurements were carried out under dark conditions at room temperature.

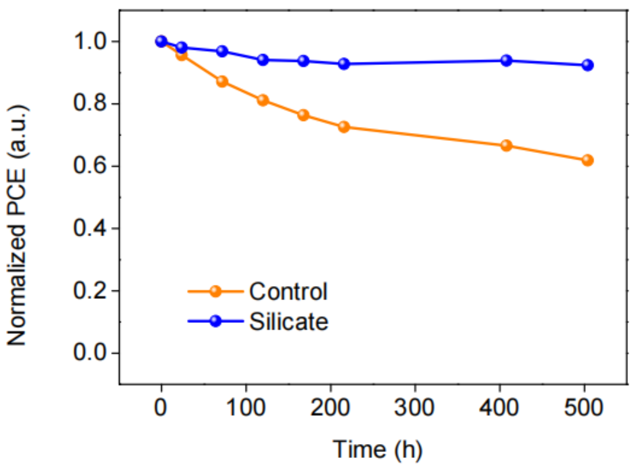

Fig. S21 Operational stability measurement of control and silicate passivated $CsPbI_2Br$ devices under continuous one-sun illumination (100 mW·cm^{-2}) in N_2-filled glovebox.

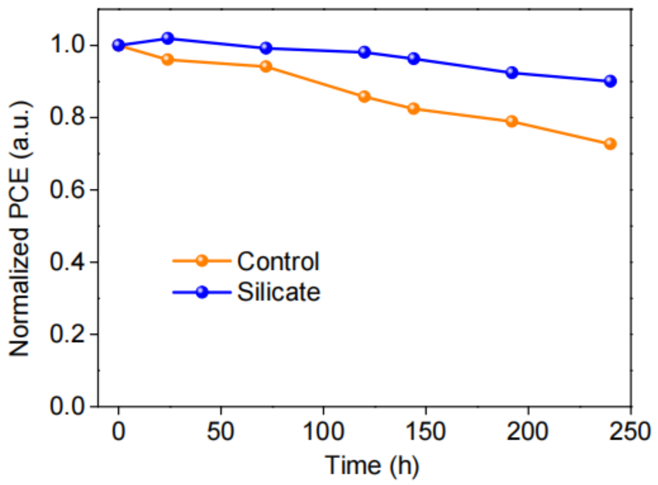

Fig. S22 Thermal stability measurement of control device and silicate passivated devices heated to 85 °C in N_2-filled glovebox

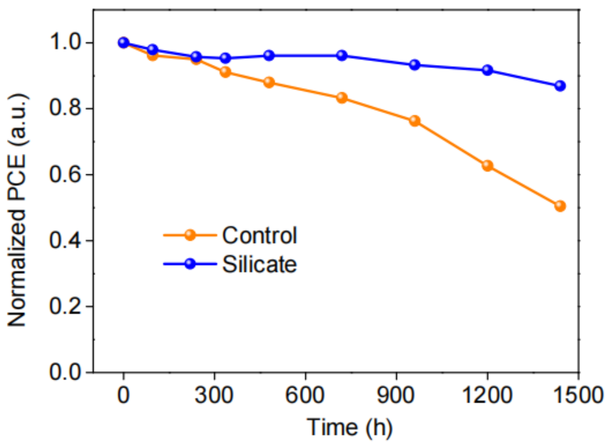

Fig. S23 Long-term stability measurements of unencapsulated PSCs in dry ambient air. The relative humidity for the stability test is ~15 ± 3%.

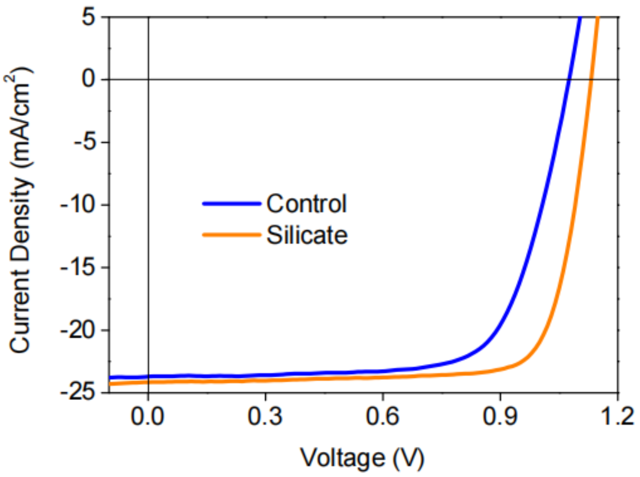

Fig. S24 J−V characteristics of the $FAPbI_3$ solar cells with and without silicate passivation measured under AM 1.5G irradiation. The device configuration is $ITO/SnO_2/FAPbI_3$/Spiro-OMeTAD/Ag. Devices were measured under simulated AM 1.5G irradiation at the reverse scan.

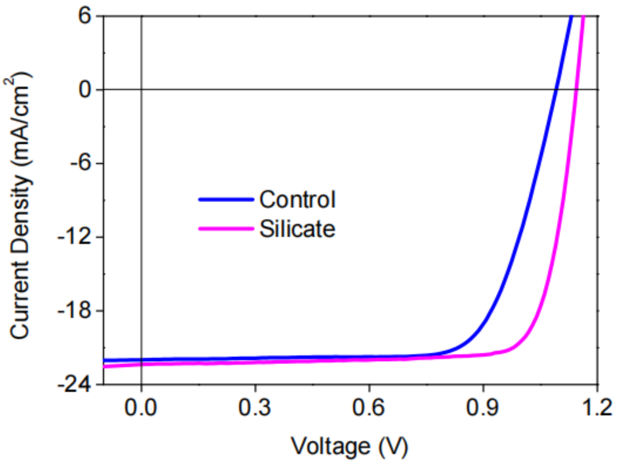

Fig. S25 J−V characteristics of the MAPbI3 solar cells with and without silicate passivation measured under AM 1.5G irradiation. The device configuration is FTO/NiOx/MAPbI3/PCBM/BCP/Ag. Devices were measured under simulated AM 1.5G irradiation at the reverse scan.

Table S1 Carrier lifetimes of CsPbI2Br films without and with oxysalt passivation obtained by fitting TRPL spectra

Samples	*A1/(A1+A2)* (%)	τ_1 (ns)	*A2/(A1+A2)* (%)	τ_2 (ns)
Control	51%	4.02	49%	15.65
Nitrate	44%	4.22	56%	20.22
Sulfate	42%	4.45	58%	26.71
Carbonate	42%	4.71	58%	30.09
Phosphate	41%	5.57	59%	30.54
Silicate	38%	5.90	62%	31.99

Table S2 Photovoltaic parameters of typical CsPbI2Br solar cells with different oxysalt passivation

Devices	Jsc (mA/cm^2)	Voc (V)	FF	PCE
Control	15.14	1.16	0.70	12.35%
Nitrate	15.41	1.19	0.74	13.61%
Sulfate	15.51	1.23	0.78	14.80%
Carbonate	15.67	1.25	0.76	14.99%
Phosphate	15.76	1.29	0.76	15.41%
Silicate	15.83	1.33	0.80	16.84%

Devices were measured under simulated AM 1.5G irradiation at the reverse scan

Table S3 Photovoltaic parameters of typical $CsPbI_2Br$ solar cells with different concentrations of silicate in SnO_2 layers

Concentration	J_{SC} (mA cm^{-2})	V_{OC} (V)	FF	PCE (%)
w/o	15.14	1.16	0.70	12.35%
0.012 M	15.36	1.24	0.75	14.33%
0.025 M	15.71	1.30	0.78	15.97%
0.05 M	15.83	1.33	0.80	16.84%
0.1 M	14.22	1.28	0.76	13.83%

Devices were measured under simulated AM 1.5G irradiation at the reverse scan.

Table S4 Comparison of the photovoltaic parameters of recently reported $CsPbI_2Br$solar cells

J_{SC} (mA cm^{-2})	V_{OC} (V)	FF	PCE (%)	Reference
15.86	1.36	0.80	17.26	This work
15.78	1.34	0.81	17.03	Nat. Commun. 2020, 11, 4237
16.34	1.334	0.801	17.46	Matter 2021, 4, 1
14.25	1.41	0.77	15.53	J. Am. Chem. Soc. 2020, 142, 9725
16.95	1.18	0.80	16.15	Nano Lett. 2019, 19, 5176.
16.82	1.15	0.75	14.69	Joule 2019, 3, 2485.
15.45	1.21	0.79	14.85	Nano Lett. 2019, 19, 6213.
15.86	1.32	0.75	15.50	ACS Energy Lett. 2019, 4, 2491.
14.00	1.28	0.78	14.00	Nat. Commun. 2019, 10, 4686.
15.30	1.30	0.81	16.20	Adv. Mater. 2019, 31, e1901152.

Supplementary References

[S1] J. He, J. Liu, Y. Hou, Y. Wang, S. Yang et al., Surface chelation of cesium halide perovskite by dithiocarbamate for efficient and stable solar cells. Nat. Commun. **11**, 4237 (2020). https://doi.org/10.1038/s41467-020-18015-5

[S2] S. Tan, T. Huang, I. Yavuz, R. Wang, M. H. Weber et al., Surface reconstruction of halide perovskites during post-treatment. J. Am. Chem. Soc. **143**, 6781-6786 (2021). https://doi.org/10.1021/jacs.1c00757
